# Willingness to receive COVID-19 vaccines, associated factors and reasons for not taking a vaccine: a cross sectional study among persons aged 13–80 years in Wakiso, Central Uganda

**DOI:** 10.1186/s12879-024-09285-1

**Published:** 2024-04-11

**Authors:** Alex Daama, Naziru Rashid, Kasango Asani, Grace Kigozi Nalwoga, Fred Nalugoda, Robert Bulamba, Emmanuel Kyasanku, Gertrude Nakigozi, Godfrey Kigozi, Joseph Kagaayi, Stephen Mugamba

**Affiliations:** 1 Africa Medical and Behavioral Sciences Organization, Nansana, Uganda; 2 Makerere University School of Public Health, Kampala, Uganda; 3 Islamic University, Mbale, Uganda; 4 Mayuge Institute of Global Health Sciences Research and Innovation, Kampala, Uganda

**Keywords:** Determinants, COVID-19 vaccine acceptance, Central Uganda

## Abstract

**Background:**

Vaccination has been recommended as one of the approaches for the control of COVID-19 pandemic. However, adequate vaccine coverage is critical to the effectiveness of the vaccine at population level. Data on acceptability of the vaccine in Ugandan urban areas are limited. This study examined the prevalence, factors associated with willingness to accept COVID-19 vaccine including reasons for not taking COVID-19 vaccine in a predominantly urban population of Wakiso, central Uganda.

**Methods:**

Data were obtained from a cross-sectional study conducted between March 1st, 2021 and September 30th, 2021 in the urban population-based cohort of the Africa Medical and behavioral Sciences Organization (AMBSO). A Multivariable modified Poisson regression analysis was used to estimate adjusted prevalence ratios (aPR) and 95% confidence intervals of willingness to accept the COVID-19 vaccine.

**Results:**

A total of 1,903 participants were enrolled in this study; 61% of whom were females. About 63% of participants indicated their willingness to accept the COVID-19 vaccine. Persons aged 13–19 years (aPR = 0.79; [95% CI: 0.74, 0.84]) or 20-29years (aPR = 0.93; [95% CI: 0.88, 0.98]) were less likely to accept the vaccine compared to persons aged 40–49 years. Persons with post-primary level of education (aPR = 1.05; [95% CI: 1.02, 1.09]) were more likely to accept the vaccine compared to persons with primary level of education. Additionally, students or individuals working in government (aPR = 1.13; [95% CI: 1.04, 1.23]) were more likely to accept the vaccine compared to individuals doing construction and Mechanic work as their main occupation. Reported reasons for not taking a COVID-19 vaccine included; concerns about side effects of the vaccine 154(57.0%), 64(23.7%) did not think the vaccines were effective, while 32(11.9%) did not like the vaccines.

**Conclusion:**

A substantial proportion of individuals were not willing to accept the COVID-19 vaccine. Health education campaigns on vaccination within urban communities could help reduce COVID-19 vaccine misconceptions in the urban populations more especially the young and persons with low levels of formal education.

**Supplementary Information:**

The online version contains supplementary material available at 10.1186/s12879-024-09285-1.

## Introduction

The Corona virus disease 2019 (COVID-19) is a respiratory illness caused by Novel Corona Virus also known as Severe Acute Respiratory Syndrome Corona Virus 2 (SARS COV-2) [1]. The control of the disease is still through preventive measures including but not limited to hand hygiene (frequent hand washing and sanitization), keeping social distance, use of face masks and vaccination [2, 3]. In Uganda, a population-based study reported a high uptake of COVID-19 preventive measures in urban settings [4]. This could potentially explain why Uganda had lower mortality rates due to COVID-19 infections compared to other countries in the world.

Globally, by 31st January 2023, 753 million COVID-19 confirmed cases and 6.8 million deaths were reported [5]. While Africa, had 9.4 million confirmed cases and 175,247 deaths, with Uganda reporting 170,233 cases and 3,630 deaths [5]. In Nigeria, between 3rd Jan 2020 and 14th June 2023, 266,675 cases and 3,155 deaths were reported [6]. Additionally, a total of 171,653 cases and 1,462 deaths were reported during the same period in Ghana [7]. Vaccination is very important in prevention and controlling the spread of infectious diseases including COVID-19 infections [8, 9]. However, for vaccination campaigns to be effective, a critical mass of the population must be vaccinated to achieve herd immunity [10–12]. Several COVID-19 vaccines have been developed for example AstraZeneca, Moderna, Pfizer, Johnson and Johnson to prevent and limit spread of COVID-19 infections [13]. These vaccines were approved and recommended for use by WHO and **Uganda like many other** countries adopted and rolled out vaccination campaigns. As of January 2023, WHO Statistics indicated that, 13 billion doses of COVID-19 vaccines had been administered globally [14]. Sadly, only 28.21% of Africa’s population had been fully vaccinated this is similar to what was observed in Uganda, indicating that only 28.34% of the Population had received at least two doses of COVID 19 Vaccines [5].

Different studies have reported varying COVID-19 Vaccine acceptance rates across the world [15–17]. A study conducted in Ethiopia among college students showed that 34.2% were willing to take up COVID-19 vaccines [18]. Another study in northwest Ethiopia reported a 55% willingness to take up COVID-19 vaccines among people with comorbidities [19]. **Furthermore**, a study conducted among students at the Islamic university in Uganda indicated that only 20.4% of the students were willing to accept COVID 19 vaccines [20]. These statistics indicate low rates of COVID-19 vaccine acceptance and uptake in Uganda and Africa at large. However, a study conducted in Canada among older persons reported a reasonably high prevalence (84%) of willingness to accept COVID-19 vaccines [21].

Potential factors that have been reported to be associated with willingness to accept COVID-19 vaccination include; gender, marital status, education level, occupation, exposure to COVID-19 infections and media such as watching television [10, 18]. Being a health care worker, having lost a loved one due to COVID-19 infections, having good knowledge about the COVID-19 vaccine, perceived susceptibility to COVID-19 infections and the effectiveness of the vaccine have also been shown to be associated with willingness to accept COVID-19 vaccines [21, 22]. A systematic review reported different reasons for vaccine hesitation or unwillingness to receive the vaccine including; concerns with vaccine safety and side effects, lack of trust for pharmaceutical industries and misinformation or conflicting information from the media [23]. Similar findings were found by a qualitative study in Germany which indicated that Low perceived benefit of vaccination, low perceived risk of contracting COVID-19, health concerns, lack of information, systemic mistrust and spiritual or religious reasons were the main reasons for not taking a COVID-19 vaccine [24]. However, this systematic review did not only include a single Ugandan study [25], but also, the said study focused on medical students leaving out the bigger population which this study addresses. The other reasons for vaccine hesitancy include; COVID-19 is not a serious illness, costs associated with the vaccine (such as office visit costs or vaccine administration fees) and distrust in COVID-19 vaccines [26–28]. However, many of these studies have been conducted in high income countries like Spain, Malaysia and USA which may not be a true representation of Uganda. Therefore, this study aimed at examining the prevalence, factors associated with willingness to accept COVID-19 vaccine and the reasons for not taking a COVID-19 vaccine in a predominantly urban population of Wakiso, central Uganda.

## Methods

### Study setting and design

This was a cross-sectional study conducted in the urban settings of Wakiso, central Uganda, in the existing Africa Medical & Behavioral Sciences Organization Population Health Surveillance (APHS) cohort that is being implemented by AMBSO. APHS is an open urban population-based cohort which enrolls about 5,000 consenting adults aged 13–80 years in the three communities of Wakiso, central Uganda and these communities include; Kazo, Lukwanga and Sentema. The AMBSO-PHS aims have been previously described through the APHS cohort profile [4, 29].

### Variables

The main variable of this study was willingness to accept COVID-19 vaccine. This was determined by asking all participants if they were willing to accept COVID-19 vaccine (Would you take the COVID-19 vaccine if it is available for your age group? “YES”/”NO”). Participants whose responses were “NO”, this was regarded as unwillingness to accept COVID-19 vaccine. We investigated reasons for not takingCOVID-19 vaccine among persons aged 13–80 years (What are your reasons for **not taking** the vaccine? ). The participants were asked different questions to assess reasons for not taking COVID-19 vaccine included; people’s concerns about side effects of the vaccines? (“‘YES/NO”), some people are not worried about getting seriously ill as a result of COVID-19 infection? (“‘YES/NO”), some people think COVID-19 vaccines are not effective? (“‘YES/NO”), some people did not like COVID-19vaccines? (“‘YES/NO”), other people are allergic to COVID-19 vaccines? (“‘YES/NO”), some people do not have time to get vaccinated? (“‘YES/NO”), other people think that they are not eligible for COVID-19 vaccines? (“‘YES/NO”), some people have other conspiracy theories? for example vaccines are not available (“‘YES/NO”). Independent variables included; age, sex, marital status, education, occupation, pregnancy status, and presence of chronic disease like HIV/AIDs, diabetes.

#### Study population

The study population included all persons aged 13–80 years in the mapped APHS communities of Wakiso, Central Uganda.

#### Inclusion criteria

A person was included in the study if him/her; was aged 13–80 years and consented to be interviewed. All participants of the APHS cohort aged 13–80 years with non-missing data on willingness to accept COVID-19 vaccine were included in the analysis.

#### Exclusion criteria

All participants who were incapacitated at the time of interview (data collection) were excluded. This is because given such situation or status could potentially limit their ability to provide informed consent to participate in the study. All participants with missing data on willingness to accept COVID-19 vaccines were also excluded from the analysis.

### Sampling procedure

This study used APHS data for round three (R3). The APHS sampling procedure has been previously described [4, 29] and this was also similar to other Population based surveys conducted elsewhere [30]. In brief, multistage sampling was employed to select the different communities. The APHS study areas were selected through community mapping exercise and comparing the population structure of areas with different distances from urban centres and cultivation [29]. The overall objective was to select representative sample of households in the different communities. In each household, all persons aged 13 years and above were considered as potential participants for the annual survey.

### Data collection

Prior to data collection, census activities were conducted. This involved household enumeration of eligible participants, obtaining information on socio-demographic characteristics such as sex, age, relationship of household members to head of the household, marital status, and residence status of each household member censused (how long have you lived in that community?). After census activities, all participants that were eligible for survey based on age (13 years and above), were then invited for a nearby venue to their household called a “hub”. This is where APHS annual survey/data collection took place, and this was done for approximately 1–2 months per community.

At a data collection hub, trained research assistants collected the data and the interviews were conducted in a private environment that suited free expression of ideas by the participants. Written informed consent was obtained before any data collection activities. The trained research assistants would first introduce themselves before consenting. Participants were given time to introduce themselves and this was done purposely to build a positive relationship with the participant and hence this improved on rapport building. Besides that, the interviews were also conducted by same sex interviewers, this increased confidence of the participants and hence they were free to open up more especially on sensitive questions. The study tools (consent forms and questionnaires) were translated into the local language (Luganda) and interviews were either conducted in Luganda (local language) or English.

The study objectives were thoroughly explained to the participants in details before obtaining their consent by signature or thumbprint. Efforts were made not to hurry the participants while maintaining a time-period of one hour and a half hour. Biological samples were drawn including anthropometrics measurements for vitals. The interviewers carried laptops to the field for data collection. The validated questionnaire was uploaded on laptops and ensured that all questions have been tackled before submitting the laptops to designated staff (editors) for editing. Upon ending the interview, the research assistants orally thanked the participants in the most appropriate language as determined by the interviewers. The editors were charged with responsibility of ensuring that there is accuracy, consistency and completeness of the data. Training and re-training of research assistants in the study protocol was done to ensure adherence with the study guidelines to avoid data collection biases.

#### Sample size

The study sample size was 1,903 participants, this was determined based on the available captured data in the APHS. However, this study would require a sample size of 509 participants using Kish Leslie formula (1965) for cross-sectional studies. A precision of 5%, standard normal deviation corresponding to 95% confidence interval (1.96), the estimated prevalence (70%) of willingness to accept COVID-19 vaccine in Uganda [31]. Considering a design effect, the sample size was multiplied by 1.5 to get 485 with a nonresponse rate of 5%.

#### Data analysis

We analyzed the data using Stata version 15 (64-bit) software. Prevalence of willingness to accept COVID-19 vaccine and vaccine hesitation were determined through a cross-tabulation. Descriptive and univariate analysis were performed to describe how the variables were distributed. The frequency distributions for categorical variables were computed. We analyzed continuous variables using mean (with the standard deviation), mode and median (with the range). Bivariate analysis was conducted between each independent factor against willingness to accept COVID-19 vaccine to determine associations. From the unadjusted analysis, variables with *p*-values < 0.2 including biologically plausible variables were included in the multivariable model. All variables with *p*-values < 0.05 were statistically significant at 95% confidence intervals for the association between factors and willingness to accept COVID-19 vaccine. We further conducted sensitivity analysis and included some of the factors that have been previously shown by other studies to be associated with willingness to accept COVID-19 vaccine. These factors included; sex, marital status, age and presence of chronic disease [32–34]. We also adjusted the cut-off *p*-value to less than 0.05 for selection of variables during multivariable analysis.

## Results

Socio-demographic characteristics and other health related factors of the study Participants. This study included 1,903 participants and the mean age of participants was 30.9 (SD = 14.0) years (Table 1). Of the 1,903 study participants, (1,170 [61.5%]) were females; (1,015 [53.4%]) were single. Most participants (984 [51.6%]) had post-primary education while, (858 [45.1%]) had primary education level, and only (61 [3.2%]) had no formal education. Occupation groups included traders (365 [19.2%]), housework (473 [24.9%]), agriculture (516 [27.1%]), construction & mechanics workers (103 [5.4%]), students or government staff (324[17.0%]) and other occupations (122 [6.4%]). About (153 [8.0%]) of participants were HIV-positive while majority of the participants (1,750 [91.96%]) were HIV negative and only 62(3.3%) of the women were pregnant.

**Table 1:** Characteristics of respondents (*N* = 1,903)

Age group	Frequencies	Percent (%)
13–19	442	23.23
20–29	631	33.16
30–39	388	20.39
40–49	211	11.09
50+	231	12.14
**Sex**		
Male	733	38.52
Female	1,170	61.48
**Marital Status**		
Married	887	46.64
Singe	1015	53.36
**Education level**		
Primary	858	45.09
Post-primary	984	51.71
Illiterate	61	3.21
**HIV results**		
Negative	1,750	91.96
Positive	153	8.04
**Pregnancy status**		
Yes	62	3.26
No	1,841	96.74

*Data presents the actual number of the survey participants. Some percentages may not total to 100% due to rounding

### Prevalence of willingness to receive COVID-19 vaccine

**Fig. 1:**
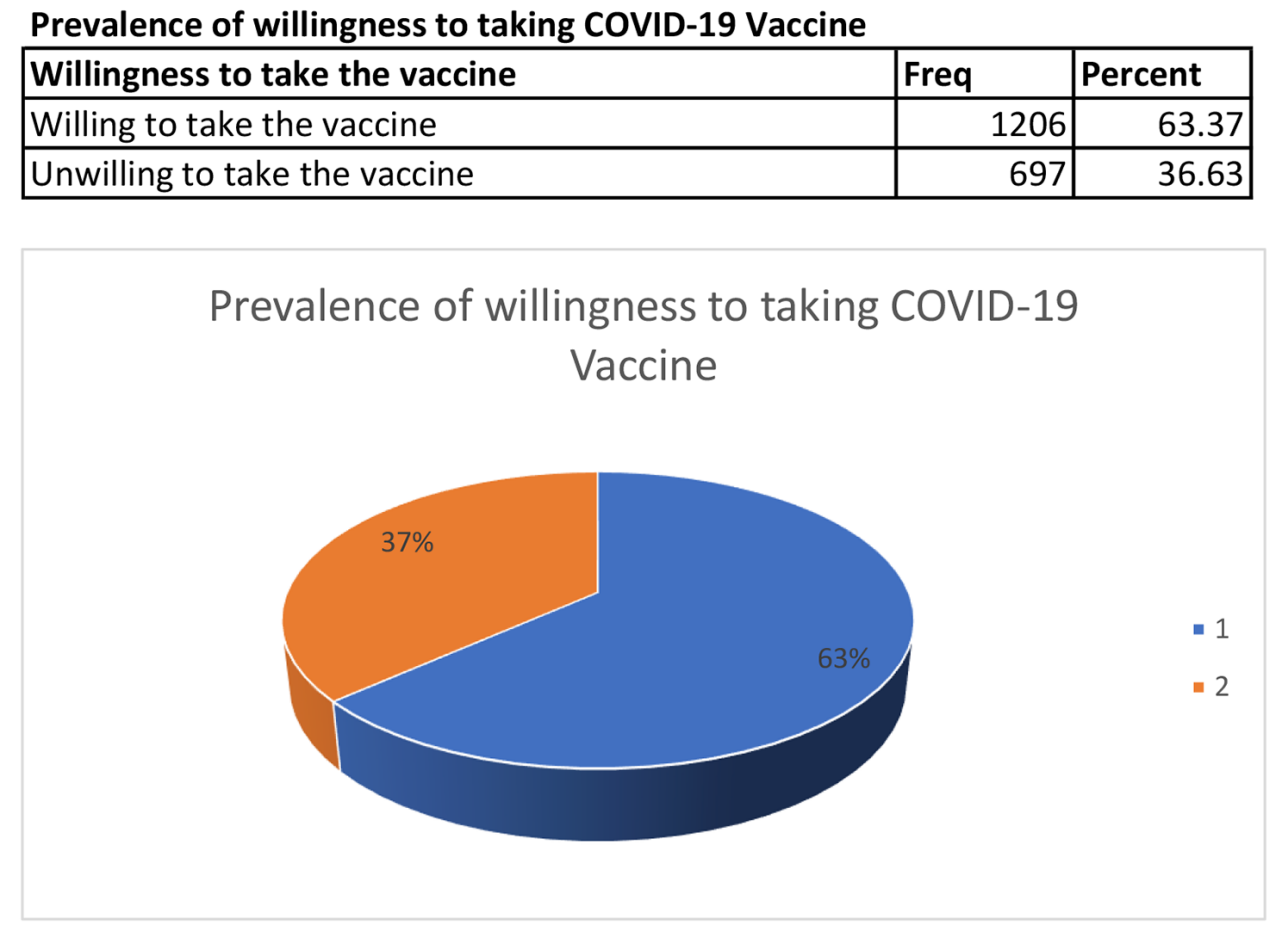
Patient selection and exclusion flow chart. Abbreviation: PC: control group; LD: low-dose group; MD: medium-dose group; HD: high-dose group; UD: ultra-high-dose group

In this study, the prevalence of willingness to accept COVID-19 was 1,206 (63.4%) while unwillingness to take COVID-19 vaccine was 697(36.6%) as shown in the Fig. 1. Of the eleven reasons for not taking COVID-19 vaccine that were reported in Table 2, majority of the participants (57%) had concerns about side effects resulting from the vaccines. About 24% of the participants did not think COVID-19 vaccines were effective, approximately 12% of the participants did not like the COVID-19 vaccines. Besides that, about 9% of the participants were not willing to accept COVID-19 vaccines because of other conspiracy theories. Additionally, about 7% of participants self-reported that they were not worried about getting seriously ill from the COVID-19 infection while less than 1% of the participants reported that long distance to the health facility or COVID-19 vaccination site was the reason for not taking a COVID-19 vaccine.

**Table 2:** Showing reasons for COVID-19 vaccine hesitancy among persons aged at least 13 years (*N* = 270)

Reasons for not taking the Vaccine	Frequencies	Percent (%)
**Concern about side effects**		
Yes	154	57.04
No	116	42.96
**Not worried about getting seriously ill from Corona virus infection**		
Yes	18	6.67
No	252	93.33
**Don’t think vaccines are effective**		
Yes	64	23.7
No	206	76.3
**Don’t like vaccines**		
Yes	32	11.85
No	238	88.15
**Allergic to vaccines**		
Yes	02	0.74
No	268	99.26
**Don’t have time to get vaccinated**		
Yes	02	0.74
No	268	99.26
**Concerns about getting corona virus infection from the vaccine**		
Yes	9	3.33
No	261	96.67
**Other conspiracy theories mentioned**		
Yes	24	8.89
No	246	91.11
**Was not eligible because had COVID 19 in the past 6 months, /pregnant, /breast feeding**		
Yes	4	1.48
No	266	98.52
**Vaccine was not available, / distance to the facility was too long**		
Yes	2	0.74
No	268	99.26
**Other reasons**		
Yes	22	8.15
No	248	91.85

*Proportion (%) among people who were not willing to receive COVID-19 vaccine (*N* = 270(14.2%) with different reasons

In a bivariate analysis (Table 3), several factors were associated with willingness to accept COVID-19 vaccine for example, participants aged 13–19 years were less likely to accept COVID-19 vaccine compared to those aged 40-49years; (cPR = 0.83; [95% CI: 0.78, 0.87]).Additionally, participants who were unmarried (cPR = 0.94; [95% CI: 0.91, 0.97]) were less likely to accept COVID-19 vaccine compared to those who were married. Participants with post-primary level of education were more likely to accept COVID-19 vaccine compared to those with primary level of education (cPR = 1.05; [95% CI: 21.02, 1.09]). Other factors that had higher likelihood of willingness to accept COVID-19 vaccine included; participants with non-formal education (cPR = 1.11; [95% CI: 1.02, 1.22]) and participants with other occupations (cPR = 1.11; [95% CI: 1.01, 1.22]). Lastly, sex, presence of chronic disease, and pregnancy status were not associated with willingness to accept COVID-19 Vaccine.

**Table 3:** Bivariable and Multivariate results showing factors associated with willingness to receive COVID-19 vaccine

Variables	Unadjusted PR	*P*-value	[95% CI]	Adjusted PR	*P*-value	[95% Cl]
Sex						
Male	1.00			1.00		
Female	1.03	0.087	0.99 1.06	1.01	0.501	0.98, 1.05
**Age group**						
40–49	1.00			1.00		
13–19	0.83	<0.001	0.78, 0.87***	0.79	< 0.001	0.74, 0.84***
20–29	0.95	0.066	0.90, 1.00	0.93	0.012	0.88, 0.98*
30–39	0.97	0.285	0.91, 1.03	0.96	0.138	0.90, 1.01
50+	1.01	0.655	0.95, 1.08	1.02	0.457	0.96, 1.09
**Educational level**						
Primary	1.00			1.00		
Post-primary level	1.05	0.001	1.02, 1.09**	1.05	0.002	1.02, 1.09**
Illiterate	1.11	0.016	1.02, 1.22*	1.08	0.097	0.99, 1.18
**Chronic diseases**						
No	1.00			1.00		
Yes	1.04	0.073	0.99, 1.09	0.98	0.42	0.93, 1.03
**Occupations**						
Construction and Mechanic workers	1.00			1.00		
Agriculturalists	1.03	0.403	0.96, 1.11	1.04	0.294	0.96, 1.13
Housework	1.08	0.056	1.00, 1.16*	1.05	0.278	0.97, 1.13
Traders/Vendors	1.05	0.247	0.97, 1.13	1.01	0.83	0.93, 1.09
Students and Govt staff	1.04	0.336	0.96, 1.12	1.13	0.005	1.04, 1.23*
Other occupations	1.11	0.027	1.01, 1.22*	1.09	0.076	0.99, 1.20

* A full adjustment method was employed, incorporating all variables, and the optimal model was determined. Prevalence ratios (PR); 95% confidence (CI); 1.00 is the reference group; significant *p*-value *p < 0.05, **p < 0.01, ***p < 0.001

In the multivariable analysis, as per the set criteria, some variables that were not significant at bivariate analysis were included in the final model and these include; comordity and pregnancy status.

In a multivariable model, young persons aged 13–19 years (aPR = 0.79; [95% CI: 0.74, 0.84]) or persons aged 20–29 years (aPR = 0.93; [95% CI: 0.88, 0.98]) were less likely to accept the vaccine compared to the persons aged 40–49 years. Interestingly, participants with post-primary level of education (aPR = 1.05; [95% CI: 1.02, 1.09]) were more likely to accept COVID-19 vaccine compared to persons with primary level of education. Additionally, students or government staff (aPR = 1.13; [95% CI: 1.04, 1.23] were more likely to accept COVID-19 vaccines compared to persons involved in construction or mechanic work. We found similar results during sensitivity analysis after adjusting for sex, presence of chronic disease and lowering the *p*-value to 0.05 when selecting variables for multivariable model (Additional file 1: Table 1).

## Discussion

About 63% of participants self-reported their willingness to accept the COVID-19 vaccine. This prevalence is slightly lower than the prevalence (72.7%) that was reported by Muhindo et al. 2022, conducted in Kampala Uganda among People living with HIV/AIDS (PLHIV) [35]. This could be attributed to the positive belief that COVID-19 vaccines are safe and beneficial to PLHIV. A phone survey conducted in Uganda reported that 91% were willing to accept COVID-19 vaccination [36]. The higher acceptance rate reported in this study that is relative to our study could be explained by the method of data collection that is to say, using phones to collect the data. Participants who owned mobile phones could have easily accessed information about COVID-19 vaccines and hence influencing their willingness to take a vaccine compared to those who did not own phones hence affecting the outcome of interest. Conversely, this study’s prevalence was higher than the prevalence of 37.3% reported by Kanyike et al., 2021 conducted in Uganda among medical students [25]. The low prevalence in this study could be attributed to the low willingness to utilize healthcare services amongst students [37]. Additionally, low proportions of willingness to accept the COVID-19 vaccine were reported in South Africa (57%) [38] and Ethiopia (54.6%) [22]. The possible explanation for the low willingness to accept COVID-19 vaccination in Ethiopia could be attributed to the differences in methodologies, our study utilized a prospective community-based approach while the Ethiopian study was a hospital-based survey. Low willingness to accept vaccination were reported in Nigeria and Ethiopia (54.6%, and 33.7%), respectively [22, 39]. The Nigerian study was conducted among the tertiary institutions compared to our study that was conducted among the general population. Therefore, this could have affected the outcome of interest (willingness to receive COVID-19 vaccine). Another study conducted in Gondor city of Ethiopia among college students reported that, only 32% of the participants were willing to receive COVID-19 vaccine [18]. Furthermore, a study conducted in South Western Ethiopia reported that, only 29% of the participants were willing to receive COVID-19 vaccine [40]. The relatively high willingness to accept COVID-19 vaccine in our study could be attributed to the vigorous campaigns by the government of Uganda to promote COVID-19 vaccination [41]. However, several studies have reported higher proportions of willingness to accept COVID-19 vaccine compared to our study findings for instance Ghana (70%) [42], Nigeria (85.29%) [43], and Canada (84%) [21]. The relatively lower prevalence of willingness to accept COVID-19 vaccine, observed in our study relative to the above studies. This could be attributed to the differences in methodologies and the socio-demographic characteristics of population studied. The overall rate of respondent’s willingness to receive COVID-19 vaccines (63%) from our findings was consistent with a systematic review. This revealed that the rate of participants’ willingness to receive the COVID-19 vaccine ranged from 27.7–91.3% [44]. We examined some of the reasons for not taking COVID-19 vaccine among persons aged 13–80 years. Majority of the participants reported concerns about side effects resulting from the COVID-19 vaccines. This finding is in line with studies conducted in Uganda and Spain [26, 45]. Besides that, our findings also revealed that vaccines were not liked and that these vaccines could instead infect them with COVID-19 virus. We found consistent findings from rural Uganda [46] and Malaysia [47]. Furthermore, our findings indicated that some people were not willing to accept COVID-19 vaccine because they had not contracted COVID-19 infection in the past six months. Therefore, they thought they were ineligible for COVD-19 vaccine uptake. This finding agrees with an online survey conducted among US adults [28]. The USA study used similar methodologies which could explain the consistency of results.

Persons aged 13–19 years (aPR = 0.79; 95% CI: 0.74, 0.84) or 20–29 years (aPR = 0.93; 95% CI: 0.88, 0.98) were less likely to accept the vaccine compared to persons aged 40–49 years. This finding is consistent with findings from studies conducted in Africa that reported that young participants were less likely to accept COVID-19 vaccine compared to older participants [40, 48–50]. The possible explanation could be that, young people perceived themselves to be safer and not at risk of COVID-19 infections as compared to the ageing population [49, 50]. We found that, persons with post-primary level of education (aPR = 1.05; 95% CI: 1.02, 1.09) were more likely to accept COVID-19 vaccine compared to those with primary level of education. This is consistent with findings from other studies conducted in other settings [18, 40, 51, 52]. The possible explanation could be that, high level of education is positively correlated with knowledge. Therefore, persons with high level of education are more likely to be knowledgeable and aware of COVID-19 preventive strategies such as vaccination compared to those with non-formal education. Our study reported that, students or government staff were more likely to accept COVID-19 vaccine (aPR = 1.13; 95% CI: 1.04, 1.23) compared to those doing construction and mechanic work as their main occupation. This finding is consistent with studies conducted elsewhere [1, 40]. The possible explanation could be attributed to ease with accessibility to information amongst government workers or students as opposed to construction/mechanic workers. Although variables such as marital status, sex and comorbidity were not associated with willingness to receive COVID-19 vaccine. These variables have been previously associated with willingness to receive COVID-19 vaccine [34, 53]. The possible explanation could be due to differences in the study methodology and socio-demographic characteristics of population studied.

### Strengths and limitations

This study is not without limitations, although the cross-sectional nature of this study was the ideal method since both the dependent and independent variables could be measured at the same time. This type of design cannot assess the temporal relationship and therefore, more advanced designs could close this gap. Additionally, this study being purely quantitative, it could not explore beliefs, attitudes and perceptions about reasons for not taking COVID-19 vaccine, hence future qualitative studies are recommended.

## Conclusion

In this study, over 30% of the population were not taking COVID-19 vaccine. Younger persons were less likely to accept COVID-19 vaccine while individuals with post-primary level of education, and students or government workers were more likely to accept the COVID-19 vaccine. The prevalence of willingness to accept the COVID-19 vaccine is still low compared to WHO recommended rate. Therefore, engaging communities through health education campaigns could support in addressing some of the reasons for not taking vaccine such as concerns about side effects of the vaccine, myths and misconceptions (COVID-19 vaccines are not effective, COVID-19 vaccines are infectious and one can easily get COVID-19 virus).

### Electronic supplementary material

Below is the link to the electronic supplementary material.

Supplementary Material 1

## Data Availability

The datasets used and/or analyzed during the current study available from the corresponding author on reasonable request.
