# Supplementary material for: Willingness to receive COVID-19 vaccines, associated factors and reasons for not taking a vaccine: a cross sectional study among persons aged 13–80 years in Wakiso, Central Uganda
Source: BMC Infect Dis. 2024 Apr 11;24:391. doi: 10.1186/s12879-024-09285-1 (PMC11008005; doi:10.1186/s12879-024-09285-1)
Supplement: Supplementary file 1 — Supplementary Material 1 [file 12879_2024_9285_MOESM1_ESM.docx]

**Additional table 1: Sensitivity analysis for multivariate Poisson regression**

| **Variables** | **Model 1 sex + presence of chronic disease+ bivariate <0.05** | | | **Model 2 bivariate <0.05** | | |
| --- | --- | --- | --- | --- | --- | --- |
|  | **Unadjusted PR** | **P-value** | **[95% CI]** | **Adjusted PR** | **P-value** | **[95% CI]** |
| Sex |  |  |  |  |  |  |
| Male | reference |  |  |  |  |  |
| Female | 1.03 | 0.087 | 0.99, 1.06 | - |  |  |
| **Marital status** |  |  |  |  |  |  |
| Married | reference |  |  |  |  |  |
| Single | 0.94 | <0.001 | 0.91, 0.97*** | 0.99 | 0.729 | 0.96, 1.03 |
| **Age group** |  |  |  |  |  |  |
| 40-49 | reference |  |  | reference |  |  |
| 13-19 | 0.83 | <0.001 | 0.78, 0.87*** | 0.79 | <0.001 | 0.74, 0.84*** |
| 20-29 | 0.95 | 0.066 | 0.90, 1.00 | 0.94 | 0.018 | 0.89, 0.99* |
| 30-39 | 0.97 | 0.285 | 0.91, 1.03 | 0.96 | 0.163 | 0.91, 1.02 |
| 50+ | 1.01 | 0.655 | 0.95, 1.08 | 1.02 | 0.472 | 0.96, 1.09 |
| **Educational level** |  |  |  |  |  |  |
| Primary | reference |  |  | reference |  |  |
| Post-primary level | 1.05 | 0.001 | 1.02, 1.09** | 1.05 | 0.002 | 1.02, 1.09** |
| Illiterate | 1.11 | 0.016 | 1.02, 1.22* | 1.08 | 0.097 | 0.99, 1.18 |
| **Comordity** |  |  |  |  |  |  |
| No | reference |  |  |  |  |  |
| Yes | 1.04 | 0.073 | 0.99, 1.09 | - |  |  |
| **Occupations** |  |  |  |  |  |  |
| Construction and Mechanic workers | reference |  |  | reference |  |  |
| Agriculturalists | 1.03 | 0.403 | 0.96, 1.11 | 1.05 | 0.221 | 0.96, 1.13 |
| Housework | 1.08 | 0.056 | 1.00, 1.16* | 1.05 | 0.197 | 0.97, 1.13 |
| Traders/Vendors | 1.05 | 0.247 | 0.97, 1.13 | 1.01 | 0.716 | 0.93, 1.09 |
| Students and Govt staff | 1.04 | 0.336 | 0.96, 1.12 | 1.14 | 0.002 | 1.04, 1.23** |
| Other occupations | 1.11 | 0.027 | 1.01, 1.22* | 1.10 | 0.056 | 1.00, 1.20* |

*Variables with a P value less than 0.05 at bivariate analysis were included in the multivariable model.
